# Telomere Dysfunction Triggers Palindrome Formation Independently of Double-Strand Break Repair Mechanisms

**DOI:** 10.1534/genetics.115.183020

**Published:** 2016-06-20

**Authors:** Vasil Raykov, Marcus E. Marvin, Edward J. Louis, Laura Maringele

**Affiliations:** * Institute for Cell and Molecular Biosciences, Newcastle University, Newcastle upon Tyne, NE2 4HH, United Kingdom; † Department of Genetics, Centre for Genetic Architecture of Complex Traits, University of Leicester, LE1 7RH, United Kingdom

**Keywords:** telomere, polymerase zeta, Rev3, Dnl4, Rad51, Rad52, palindrome

## Abstract

Inverted chromosome duplications or palindromes are linked with genetic disorders and malignant transformation. They are considered by-products of DNA double-strand break (DSB) repair: the homologous recombination (HR) and the nonhomologous end joining (NHEJ). Palindromes near chromosome ends are often triggered by telomere losses. An important question is to what extent their formation depends upon DSB repair mechanisms. Here we addressed this question using yeast genetics and comparative genomic hybridization. We induced palindrome formation by passaging cells lacking any form of telomere maintenance (telomerase and telomere recombination). Surprisingly, we found that DNA ligase 4, essential for NHEJ, did not make a significant contribution to palindrome formation induced by telomere losses. Moreover *RAD51*, important for certain HR-derived mechanisms, had little effect. Furthermore *RAD52*, which is essential for HR in yeast, appeared to decrease the number of palindromes in cells proliferating without telomeres. This study also uncovered an important role for Rev3 and Rev7 (but not for Pol32) subunits of polymerase ζ in the survival of cells undergoing telomere losses and forming palindromes. We propose a model called short-inverted repeat-induced synthesis in which DNA synthesis, rather than DSB repair, drives the inverted duplication triggered by telomere dysfunction.

TELOMERES are DNA and protein complexes that help distinguish chromosome ends from double-strand breaks (DSBs), thus preventing their inadvertent repair. The average telomere length decreases with age. This is because most human somatic cells have insufficient telomerase activity, required to counterbalance the telomere losses during DNA replication. Telomeres shorten prematurely in certain diseases and genetic syndromes, for example, liver cirrhosis, pulmonary fibrosis, and dyskeratosis congenita (Alder *et al.* 2011; Batista *et al.* 2011; El-Chemaly *et al.* 2011). Moreover, telomeres can be lost completely, as in progenies of human lymphocytes exposed to heavy ions, or during chromothripsis (Durante *et al.* 2006; Štafa *et al.* 2014).

Telomere losses may trigger formation of chromosomal deletions and duplications, including palindromes, thus contributing to loss of genomic guardians or amplification of oncogenes. Consistent with this hypothesis, palindromes are often detected in human cancer cells (Tanaka *et al.* 2005; Guenthoer *et al.* 2012) and their frequency increases with telomere dysfunction in mouse cancer cells (O’Hagan *et al.* 2002). Moreover, palindromes are found near telomeres in syndromes with severe mental retardation (Zuffardi *et al.* 2009) and in autism (Devillard *et al.* 2010). When localized near telomeres, palindromes are seen as telomere fusions, triggered by telomere dysfunction and caused by the nonhomologous end-joining (NHEJ) pathway of DSB repair (McEachern *et al.* 2000; Lo *et al.* 2002). However, when palindromes are experimentally triggered by DSBs and DNA replication defects, their formation appears to require homologous recombination (Mizuno *et al.* 2009; Brewer *et al.* 2011) or its variants: the single-strand annealing (VanHulle *et al.* 2007), break-induced replication (Butler *et al.* 2002; Rattray *et al.* 2005) and intramolecular recombination (Butler *et al.* 1996; Tanaka *et al.* 2002). A role for NHEJ or homologous recombination in the spontaneous formation of palindromes triggered by telomere losses has not been studied yet.

An excellent model system to study palindrome formation triggered by telomere losses is the budding yeast PAL system (Maringele and Lydall 2004b). PAL strains are lacking both the *TLC1* and *RAD52* genes, essential for telomere elongation by telomerase or by recombination, and therefore lacking any means to maintain telomeres. In consequence, PAL cells undergo telomere attrition, leading to a permanent state of cell cycle arrest called replicative senescence, similarly to human somatic cells undergoing telomere dysfunction. However, some PAL cells are able to escape senescence and proliferate indefinitely (Maringele and Lydall 2002). After several population doublings, deletions and palindromes appear on chromosome ends of these cells. Palindromes formed at inverted repeats near chromosome ends are essential for survival of cells proliferating with lost telomeres. This has been experimentally demonstrated by monitoring the progressive end-chromosomal degradation characteristic of cells lacking telomeres, by Southern blotting (Maringele and Lydall 2004b; Maringele and Lydall 2005). When the chromosome degradation approached essential genes (for example *BRR2* on chromosome 5), signals indicative of chromosome ends were completely replaced by signals indicative of palindromes, thus demonstrating that cells failing to form palindromes perish from the population (Maringele and Lydall 2004b; Maringele and Lydall 2005). The mechanism(s) responsible for palindrome formation triggered by telomere losses remains unclear.

In this study, we used the PAL system to investigate a role for DSB repair mechanisms in palindrome formation triggered by telomere losses. Surprisingly, we found that the majority of end-chromosomal palindromes were not dependent upon telomere fusions, *e.g.*, NHEJ. Moreover, the end-chromosomal palindrome formations induced by telomere losses were inhibited, rather than facilitated by homologous recombination (HR). Our experiments also revealed an unexpected essential role for Rev3, the catalytic subunit of polymerase ζ, in facilitating escape from senescence of cells lacking telomeres and forming palindromes. This observation was supported by a similarly important role for Rev7, a polymerase ζ subunit/cofactor. This role of polymerase ζ is in strong contrast with those of polymerase ε Dpb3 and Dpb4 cofactors, which were found to inhibit escape from senescence. Pol32, a subunit of both polymerase ζ and Δ, had a neutral effect. We propose a NHEJ- and HR-independent model of palindrome formation, called short-inverted repeat-induced synthesis (SIRIS), in which the DNA synthesis plays the major role.

## Materials and Methods

### Yeast strains

All strains used in this study are in the W303 background and *RAD5^+^*. Since W303 strains contain an *ade2-1* mutation, YPD medium was routinely supplemented with adenine at 50 mg/liter. All PAL strains originated from a diploid heterozygous for the following genes: *TLC1/tlc1*∆::*HIS3*, *RAD52/rad52*∆::*TRP1*, and *EXO1/exo1*∆::*LEU2*. In this diploid, we knocked out genes by converting them into G418-MX cassettes (Longtine *et al.* 1998). Diploid cells were sporulated and haploids selected by random spore analysis. Then, 20 haploids for each genotype were tested by PCR to reconfirm the deletion of genes of interest and passaged on YPD plates at 25°, initially every second day till they enter senescence, then every 4–5 days by pooling colonies on a toothpick (about 1 × 10^7^ cells) and streaking them onto fresh plates.

### NHEJ assay

The PRS416 centromeric vector was linearized with *Not*I for 2 hr at 37°, then *Not*I was inactivated at 60° for 20 min. Approximately 2.5 × 10^7^ cells in stationary phase (*e.g.*, maintained for 5 days at 4°) were transformed by the lithium acetate-based method with 1 μg vector (either *Not*I-cut or uncut/circular). Transformed cells were plated onto selective plates and incubated at 25°. Colonies were counted after 4–5 days.

### Telomere and chromosome V Southern blotting

Telomere blotting was performed as previously described (Maringele and Lydall 2004a). Briefly, ∼20 ng of genomic DNA was digested with *Xho*I and separated on a 0.8% agarose gel. DNA was transferred to a Magna Nylon membrane (Genetic Research Instrumentation) and UV cross-linked. The membrane was then hybridized with a fluorescein-labeled probe consisted of 120 bases of telomeric thymine-guanine (TG) sequences (obtained by PCR using pDL912 as a template). Hybridization was detected according to Amersham protocols. Chromosome V Southern blotting was performed exactly as described (Maringele and Lydall 2004b).

### Comparative genome hybridization

Microarray probes (40–70 mer) representing 6250 ORFs in the *Saccharomyces cerevisiae* genome were purchased from Eurofins (Lancaster, PA). These were printed onto aldehyde^+^ slides (Genetix) using an in-house arrayer. Sample and reference DNA were random labeled using a BioPrime Array CGH Genomic Labeling Module (Invitrogen, Carlsbad, CA) and Cy5, Cy3-conjugated deoxyuridine 5-triphosphate (dUTP) (Amersham, Piscataway, NJ). The efficiency of each labeling reaction was quantified using Nanodrop ND-1000 and 50 pmol of labeled target material was competitively hybridized to arrays for at least 18 hr at 62° using M-Series Lifterslips (Erie Scientific). Following washes, arrays were immediately scanned and analyzed using Genepix 6 and a 4000B reader (Axon Instruments). Scanned images were then analyzed and spots of irregular shape containing high background or hybridization artifacts were flagged and omitted from further analysis. Data were then normalized using ratio-based normalization, so that the mean of the ratio of medians was equal to one. Data were then exported into Aquity 4.0 for further analysis. Unlogged median of PAL/WT ratio values was used to draw chromosome plots in Acuity 4.0 using Caryoscope mode. ORFs with a ratio between 0.01 and 0.5 were considered deleted, while ORFs with a ratio between 1.5 and 2.5 were considered duplicated. To avoid artifacts, we considered a chromosomal region to be amplified when at least three adjacent ORFs had ratio values of at least 1.5.

### Data availability

The authors state that all data necessary for confirming the conclusions presented in the article are represented fully within the article.

## Results

### Cells proliferating without telomeres are NHEJ proficient

One of the classical roles attributed to telomeres is to prevent chromosomes from fusing together or circularizing. This is because in the absence of functional telomeres, NHEJ factors detect chromosome ends as DSBs and proceed with their fusion (Liti and Louis 2003). Fusion of dysfunctional telomeres is eventually followed by breakage of the resulting dicentric chromosomes and loss of viability. Therefore, the ability of PAL cells to proliferate indefinitely after losing telomeres is very intriguing (Maringele and Lydall 2004b).

**Figure 1:**
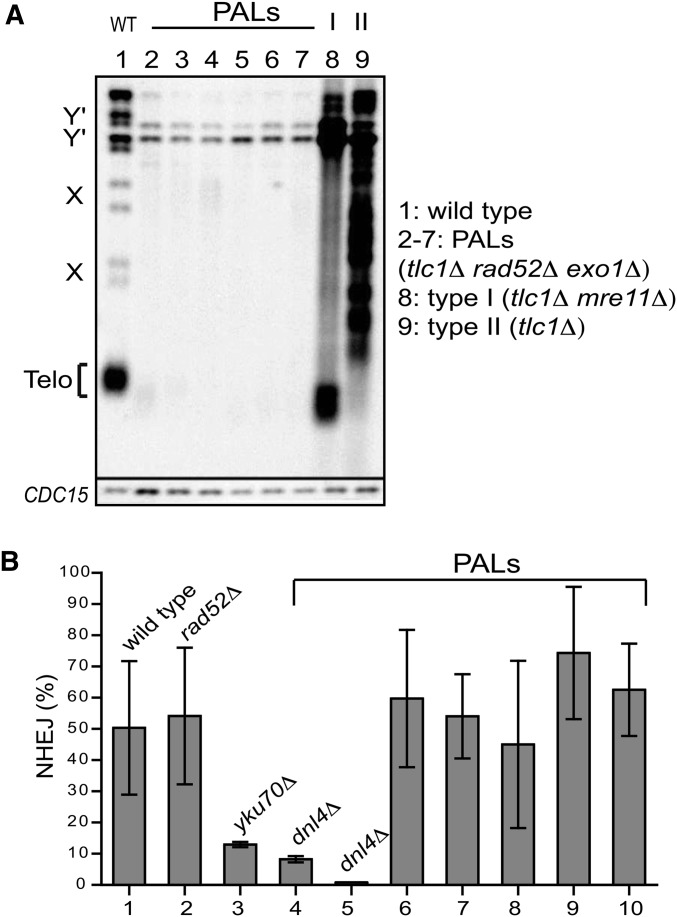
Cells proliferating without telomeres are NHEJ proficient. (A) Telomere blotting of restriction fragments corresponding to Y′ subtelomeres (Y′) and telomeres (TELO). Lane 1 shows the WT. Lanes 2–7 show *tlc1*∆ *rad52*∆ *exo1*∆ strains (*e.g.*, PAL strains, passage 60). Lane 8 shows a type I survivor (*e.g.*, a *tlc1*∆ *mre11*∆ strain with amplified subtelomeres). Lane 9 shows a type II survivor (*e.g.*, a *tlc1*∆ strain with amplified telomeres). The *CDC15* gene was detected as a loading control. (B) PAL and control strains were transformed with a centromeric vector, which was either cut (linearized) or left intact (circular). Columns represent the NHEJ efficiency, *e.g.*, the fraction of colonies obtained after transformation with the linear vector, relative to that obtained with the circular vector. Relevant mutations are indicated above each column. Error bars represent the standard deviation from three independent experiments.

One possible explanation for the long-term survival of PAL strains is that they inactivate the NHEJ pathway. To test this hypothesis, we generated and propagated several PAL strains (*e.g.*, *tlc1*∆ *rad52*∆ *exo1*∆ haploid strains) as previously described (Maringele and Lydall 2004b). Exo1 is an exonuclease that degrades chromosome ends lacking telomeres (Xue *et al.* 2016). We confirmed that PAL survivors have lost the telomeric sequences (Figure 1A, lanes 2–7) in contrast to the HR-dependent survivors, called type I and II (Figure 1A, lanes 8 and 9). To investigate whether PAL strains were NHEJ proficient, we transformed PAL cells in the G1 phase with either a cut (linearized) or an uncut (circular) vector. Colonies were counted on selective plates and the NHEJ capacity was calculated as the percentage of colonies formed after the transformation with the linearized vector, relative to those transformed with the circular vector. We found that all analyzed PAL strains were NHEJ proficient, being able to ligate DNA ends similarly to the WT and *rad52*∆ strains (Figure 1B). In contrast, PAL strains with additional *dnl4*∆ or *yku70*∆ mutations, lacking factors essential or important for NHEJ (*e.g.*, the DNA ligase 4 or the Yku70 part of the KU complex, respectively) were largely unable to ligate DNA (Figure 1B). These data indicate that PAL strains were NHEJ proficient, unless we deliberately inactivated relevant NHEJ factors.

### Chromosome end duplications form independently of NHEJ

The ability of PAL strains to proliferate long term, despite undergoing a progressive chromosome shortening, was proposed to be facilitated by DNA palindromes (Maringele and Lydall 2004b; Lee *et al.* 2008). This is because palindromes could prevent the loss of essential genes, since essential and other genes become duplicated at distance from the continuously eroding chromosome ends. One plausible hypothesis explaining formation of palindromes in strains proliferating without telomeres, is that NHEJ fuses sister chromatids together, thus generating dicentric chromosomes. Dicentrics can break asymmetrically during mitosis, when pulled toward opposite spindle poles, in which case one cell gets a chromosome with a palindrome, whereas the other cell gets a chromosome with a deletion. To test this hypothesis, we generated PAL strains lacking the *DNL4* gene, encoding the DNA ligase 4, essential for NHEJ.

**Figure 2:**
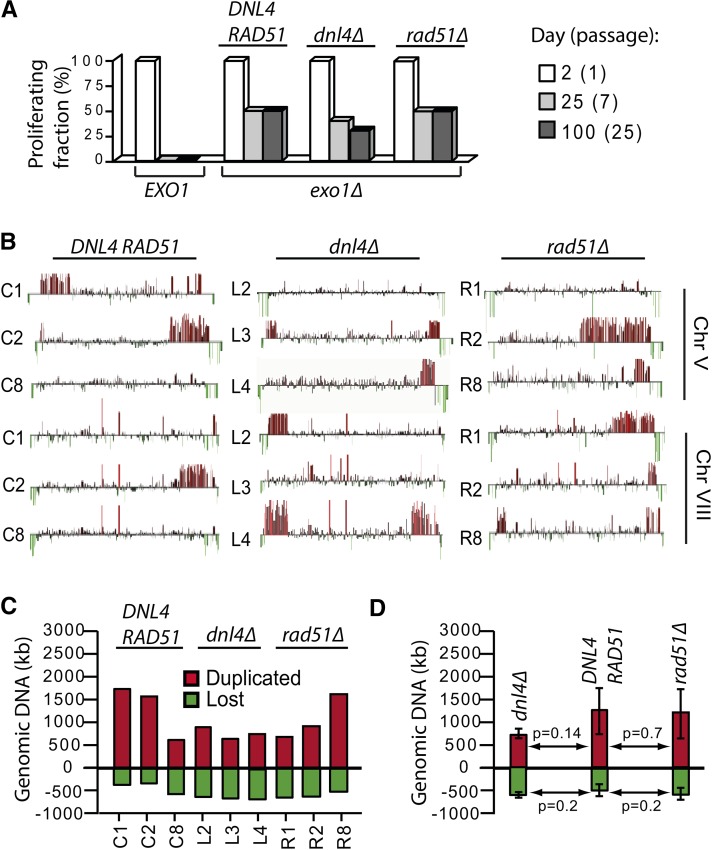
The effect of *DNL4* and *RAD51* in PAL strains. (A) Numerous independent *tlc1*∆ *rad52*∆ *exo1*∆ strains ± other mutations (indicated above the columns) were propagated for ∼25 passages. Indicated by columns is the fraction of strains proliferating: at passage 1 (white); at passage 7, *e.g.*, the fraction of strains escaping senescence (light gray); and at passage 25 (dark gray). (B) CGH analysis of chromosomes V and VIII in three independent PAL strains. Spikes above the baseline show gene duplications; spikes below the baseline show gene deletions. All strains are *tlc1*∆ *rad52*∆ *exo1*∆ (± other indicated mutations). (C) Columns above the baseline show the total amount of duplicated DNA in each strain; columns below the baseline show the total amount of deleted DNA. Strain numbers are indicated below the columns and relevant genotypes above the columns. (D) Statistical analysis of the data shown in C. The *P*-value was calculated using the unpaired *t*-test (http://www.graphpad.com). The error bars indicate the standard deviation.

Numerous *dnl4*∆ and *DNL4^+^* PAL strains were propagated on plates. About half of them were able to escape senescence and resume proliferation, unless cells were *EXO1^+^*, consistent with previous reports (Maringele and Lydall 2004b). We found that similar fractions of *dnl4*∆ and *DNL4^+^* PAL strains were able to escape senescence and proliferate for at least 50 passages, *e.g.*, 200 days (Figure 2A). A small decline in the proliferation fraction was observed for *dnl4*∆ PAL strains only, which could be due to their inability to repair spontaneous DSBs. Another explanation could be that *dnl4*∆ PAL strains are deficient in palindrome formation, and therefore will eventually perish due to loss of essential genes. Therefore, we compared the ability of *dnl4*∆ and *DNL4^+^* PALs to form chromosome duplications by comparative genomic hybridization (CGH).

We found similar numbers of duplications in *dnl4*∆ and *DNL4^+^* PAL strains (Figure 2B). Moreover, we quantified the total amount of duplicated and deleted chromosomal DNA for each PAL strain (Figure 2C). We found that *dnl4*∆ have duplicated in average slightly less DNA than *DNL4^+^* PAL strains; however, the difference was not statistically significant (*P* = 0.14, Figure 2D). Furthermore, there was no significant difference between the amounts of DNA lost (*e.g.*, the sum of all terminal deletions) in *dnl4*∆ *vs.* *DNL4^+^* PAL strains (*P* = 0.2). These data indicate that a *dnl4*∆ mutation did not affect the chromosomal duplication or deletion triggered by telomere losses. We concluded that NHEJ and telomere fusion play little role in generating the end-chromosomal duplications and deletions found in strains proliferating without telomeres.

### Telomere losses trigger terminal deletions and duplications irrespective of Rad51

PAL strains are defective in HR, due to the absence of Rad52. Deletion of *RAD52* was necessary for preventing the amplification of telomeres or subtelomeres. However, Rad52-independent types of HR exist, and they depend upon Rad51 (Coic *et al.* 2008). Moreover, Rad51 is essential for HR in mice, chicken, and fission yeast, reviewed by Octobre *et al.* (2008). Therefore, we asked whether a Rad51-dependent mechanism was responsible for the formation of palindromes. To investigate a possible role for Rad51, we compared the ability of *rad51*∆ *vs.* *RAD51^+^* PAL strains to proliferate long term and generate chromosomal duplications, during similar experiments to those described for *DNL4* (Figure 2). We found that escape from senescence and proliferation of *rad51*∆ PAL survivors was indistinguishable from those of the *RAD51^+^* homologs (Figure 2A). Moreover, the number of duplications detected by CGH was similar in *rad51*∆ and *RAD51^+^* PAL strains (Figure 2B). Furthermore, the amount of deleted or duplicated genomic DNA was also similar in these strains (Figure 2, C and D). We conclude that Rad51 is not involved in generating terminal duplications and deletions in strains proliferating without telomeres.

### Genomic duplications triggered by telomere losses are palindromes

**Figure 3:**
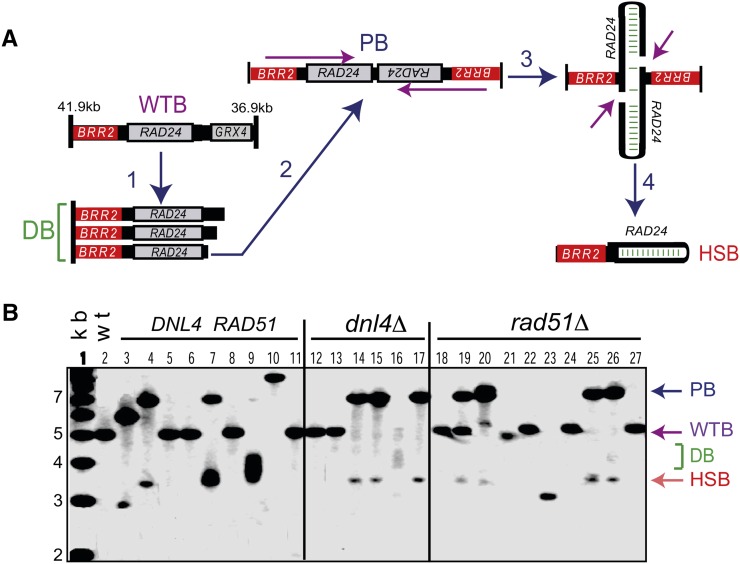
Chromosomal duplications triggered by telomere attrition are palindromes. (A) Cartoon explaining the succession of events and the nature of the DNA fragments detected during palindrome formation on the right arm of chromosome V. (1) DNA loss approaches the essential gene *BRR2* and therefore the end of the chromosome is detected as a diffuse resection band (RB) close to *BRR2*. (2) Palindromes form at a certain IR hot spot, in which case a 7-kb PB is detected. (3 and 4) Some of the palindromes are processed by resolvase, generating a HSB. Alternatively, they are forming hairpins. (B) Southern blotting analysis of DNA extracted from WT (lane 2) and from 25 independent PAL strains (lanes 3–27). Lane 1 contains the molecular weight marker. Additional relevant mutations are indicated above the lanes. Normal chromosomal regions give 5-kb WTBs. Progressive loss of DNA up to the hotspot gives a lower diffuse band (DB). Formation of a palindrome at the hotspot gives a 7-kb PB and a 3.5-kb HSB.

Palindromes are difficult to distinguish from other types of duplication, and also notoriously difficult to sequence. However, palindromes form secondary structures, and some of them are processed *in vivo* by specialized enzymes (Eichman *et al.* 2000), resulting in two fragments that can be identified by Southern blotting as half-sized bands (HSBs), the “signature” of a palindrome (Maringele and Lydall 2004b). To determine whether duplications detected by CGH were palindromes, we investigated the right arm of chromosome 5. This arm contains a hotspot for palindrome formation (*e.g.*, a 12-bp AT-rich inverted repeat), situated near the essential gene *BRR2*, as previously described (Maringele and Lydall 2004b). The nature of the bands detected by Southern blotting is described in Figure 3.

We found that three of nine investigated *DNL4^+^ RAD51^+^* PAL strains formed palindromes on the right arm of chromosome 5 after 50 passages, indicated by the codetection of palindrome bands (PBs) and HSBs (Figure 3B, lanes 3, 4, and 7). PAL strains analyzed in lanes 3 and 4 show differently sized PB and HSB, indicating that palindromes initiated at different hot spots on chromosome 5. The PAL strain in lane 10 shows a very high band with no HSB; therefore this band may indicate a translocation, rather than a palindrome. Moreover, three of six investigated *dnl4*∆ PAL strains formed palindromes, initiated at the AT-rich hotspot (Figure 3B, lanes 14, 15, and 17). Furthermore, 4 of 10 analyzed *rad51*∆ PALs formed palindromes, initiated at the same hotspot (lanes 19, 20, 25, and 26).

Several strains analyzed in Figure 3B have also been analyzed by CGH. Palindromes detected in lanes 3, 4, 14, 15, 19, and 25 (Figure 3B) corresponded to genomic duplications detected by CGH, whereas WT-like bands (WTBs) and unidentified bands (Figure 3B, lane 10 and 23) did not. We conclude that genomic duplications formed in strains lacking Rad51 and DNA ligase 4 and proliferating without telomeres were palindromes.

### Early PAL survivors convert to type I survivors when transformed with *RAD52*

It is clear that palindromes triggered by telomere losses can form in the absence of Rad52, since all PAL cells have the *rad52*∆ mutation. However, Rad52 plays an essential role in many models of palindrome formation triggered by DSBs. Therefore, we hypothesize that many more palindromes could have formed in PAL strains, if they were *RAD52^+^*. To test this hypothesis, early- and late-passage PAL strains (*e.g.*, propagated for 10 and 50 passages, respectively) were transformed with an “empty” vector, with the *TLC1* gene (encoding the catalytic subunit of telomerase) or with the *RAD52* gene. A few colonies were random picked from the transformation plates and propagated under selective pressure, for another 25 passages.

**Figure 4:**
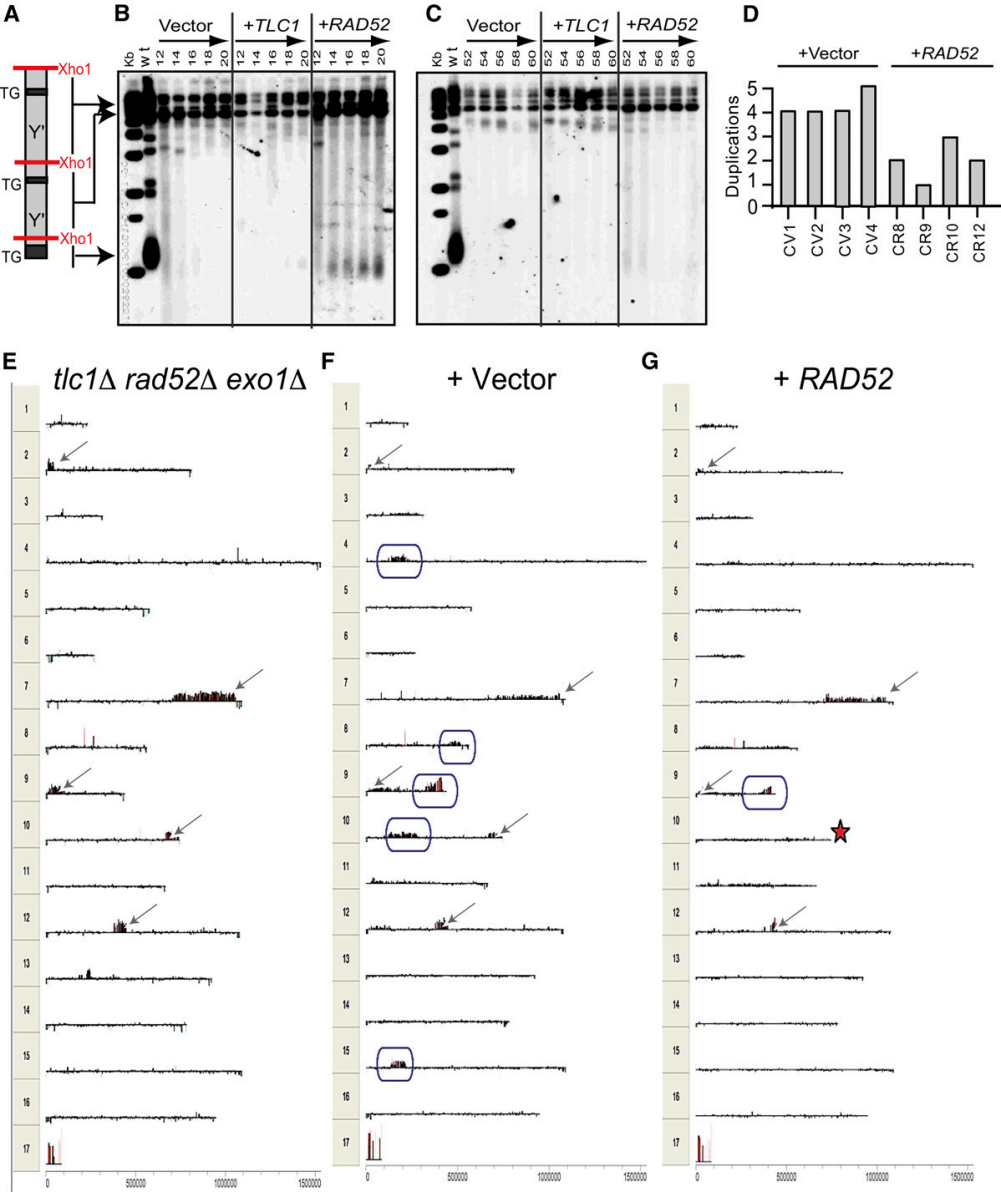
Genomic changes induced by *TLC1* and *RAD52* in PAL strains. PAL strains were transformed with a vector carrying the *TLC1* or *RAD52* gene (as indicated above the Southern blots). We randomly picked a few colonies from the transformation (−URA) plates and passaged them further under selective pressure. (A) Representation of a chromosome end cut with *Xho*1. (B) Telomere blotting of an early-passage PAL strain, transformed with an empty vector, with *TLC1*, or with the *RAD52* gene. Passage numbers and genes are indicated above lanes. First two lanes show the molecular weight marker and the WT. (C) As in B, except that a late-passage strain was transformed. (D) Columns indicate the number of chromosomal duplications detected in several transformants of a late-passage strain, after 25 passages, since its transformation with either an empty vector, or with *RAD52*. The unpaired *t*-test showed statistically significant differences (*P* = 0.003) between the two groups. (E) CGH analysis of a late-passage PAL strain, just before its transformation with an empty vector or with *RAD52*. Spikes above the baseline indicate gene duplications, below the baseline, gene losses. Numbers 1–16 indicate chromosomes and 17, the mitochondrial DNA. Arrows are pointing to duplications present at the time of the transformation. (F) As in E, except that we analyzed an empty vector transformant, 25 passages after the transformation. New duplications (*e.g.*, not found in E) are in-frames. (G) As in F, except that we analyzed a *RAD52* transformant. Duplications found in E but not in G are marked by a star.

We found that subtelomeres of early-passage PAL strains transformed with *TLC1* remained indistinguishable from those of PAL strains transformed with an empty vector (Figure 4, A and B). These data indicate that transformation with telomerase had little effect in PAL strains, most likely due to erosion of the TG sequences. In contrast, transformation with *RAD52* was followed by the amplification of subtelomeric Y′ regions in early passage PAL strains (Figure 4B), but not in late-passage PAL strains (Figure 4C). In conclusion, early- but not late-passage PAL strains convert to type I survivors, upon their transformation with *RAD52*. Therefore, PAL strains are losing the ability to convert over time. This is most likely due to insufficient homology (required for a Rad52-dependent homologous recombination) between chromosome ends with partially or completely lost subtelomeres.

### Rad52 protects against palindromes triggered by telomere losses

Since late-passage PAL strains did not convert to type I survivors following their transformation with *RAD52*, we tested by CGH whether more palindromes formed in these strains than in control strains. We found that this was not the case. In fact, fewer palindromes were detected after 25 passages post-transformation with *RAD52* than with the empty vector, and the difference was statistically significant (Figure 4D). For example, Figure 4E shows the CGH analysis of a late-passage PAL strain prior to its transformation. Five duplications were detected (indicated by arrows). In colonies taken from the transformation plate and propagated for another 25 passages under selective pressure, new duplications were detected: five in cells transformed with the empty vector (indicated by circles, Figure 4F), but only one in cells transformed with *RAD52* (Figure 4G). Moreover, the *RAD52^+^* cells appear to have lost one of the duplications detected prior to their transformation (marked with a star on chromosome 10, Figure 4G). These data indicate that Rad52 and HR do not contribute to the formation of palindromes in strains proliferating without telomeres. In contrast, HR appears to protect against palindromes triggered by telomere losses.

### Polymerase ζ is required for the long-term proliferation of PAL strains

**Figure 5:**
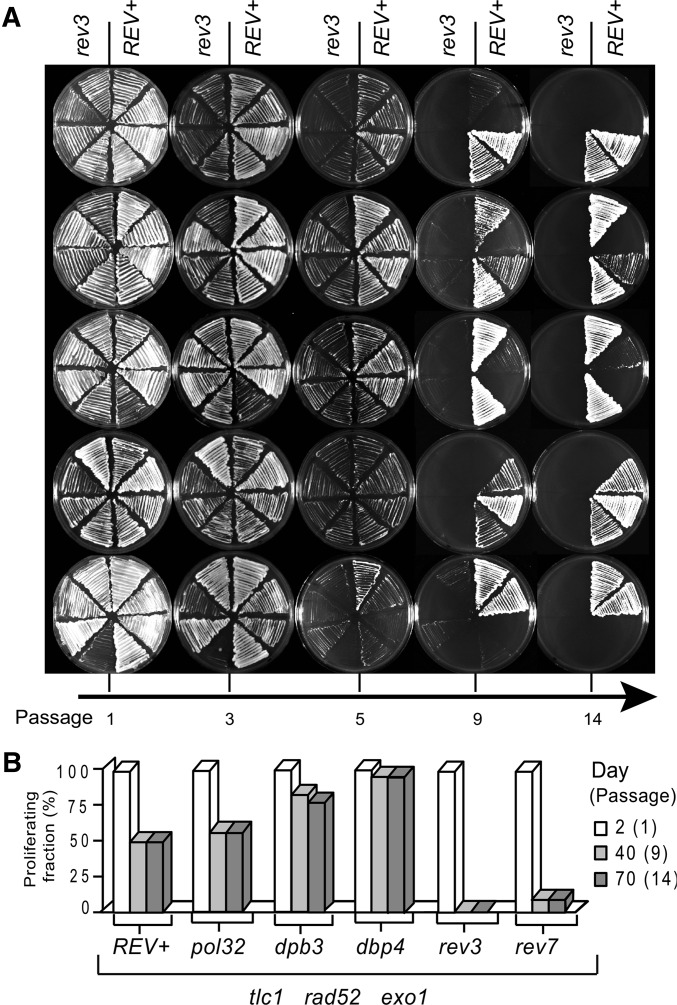
Rev3 and Rev7 are important for proliferation of PAL strains. All strains are *tlc1*∆ *rad52*∆ *exo1*∆. (A) Several newly germinated *rev3*∆ strains (left) and the same number of *REV^+^* controls (right) were propagated on a succession of plates and photographed at the indicated passage. (B) Columns represent the fraction of PAL strains propagated on plates as in A and proliferating up to the indicated passage. Relevant mutations are indicated below columns.

Palindrome formation requires DNA synthesis. To identify DNA polymerases involved in palindrome formation, we tested whether subunits of polymerase ζ, Δ, or ε affected the long-term proliferation of PAL strains. We generated numerous PAL strains lacking *REV3* or *REV7* (encoding the catalytic and the accessory subunit of the translesion polymerase ζ, respectively), lacking *POL32* (encoding a subunit of polymerases Δ and ζ), or lacking *DPB3* or *DPB4* (encoding subunits of polymerase ε) and propagated them on plates, together with controls. An example of this proliferation assay is shown in Figure 5A.

Interestingly, we found that all tested *rev3*∆ PAL strains were incapable of long-term proliferation (*e.g.*, beyond the 5th passage, when they were senescent, Figure 5A) Similarly, most *rev7*∆ PAL strains perished, with only 10% proliferating long term, whereas ∼50% of *REV^+^* PAL strains proliferated (Figure 5B). In contrast, many more *dpb3*∆ or *dpb4*∆ PAL strains escaped from senescence (∼80–90%), whereas the fraction of *pol32*∆ escapers was only marginally higher than that of controls (Figure 5B), consistent with a previous study (Deshpande *et al.* 2011). Almost all of PAL strains that escaped from senescence were able to proliferate long term (Figure 5B), similarly to the PAL strains analyzed in Figure 2, suggesting that they had similar abilities to prevent the loss of essential genes by forming palindromes. In conclusion, Rev3 and Rev7 are required for the long-term proliferation of PAL strains, whereas the Dpb3, Dpb4, and Pol32 subunits are not.

## Discussion

Palindromes detected in eukaryotic cells with telomere defects were attributed to the DSB repair mechanisms, particularly the NHEJ variant called sister chromatid fusion (SCF), leading to formation of dicentric chromosomes and breakage–fusion–bridge (BFB) cycles (Lo *et al.* 2002). However, palindromes detected in telomerase-defective *Caenorhabditis elegans* were not consistent with this mechanism (Lowden *et al.* 2011). Our study shows that palindromes triggered in response to telomere losses in budding yeast are also inconsistent with SCF and BFB mechanisms. First, several palindromes are stretching over the centromeric regions (Supplemental Material, Figure S1) and therefore are unlikely to have formed during dicentric breakage, since this type of breakage happens in-between centromeres pulled in opposite directions. Moreover, palindrome formation was independent of DNA ligase 4, essential for SCF. Plausible explanations could be that yeast cells undergoing SCF are incapable of long-term proliferation, or that SCF events are prevented by proteins like NEJ1 (Liti and Louis 2003) or by an excessive resection of chromosome ends.

Another pathway that results in SCF is the single-strand annealing pathway (SSA). This is a HR variant, detected in connection with direct or inverted repeats in response to a DSB in budding yeast (VanHulle *et al.* 2007) or to rapidly degraded telomeres in fission yeast (Wang and Baumann 2008). However, SSA (and other mechanisms for which Rad52 or Rad51 are important) played surprisingly little role in generating palindromes in strains proliferating without telomeres. Contrary to what was expected, *RAD52^+^* PAL strains appeared to form fewer chromosomal duplications than *rad52*∆ PAL strains. This could be explained by recombination events involving palindrome arms, leading to loss of palindromes, for example, the loss of the palindrome on chromosome 10 (Figure 4, E and F *vs.* G).

**Figure 6:**
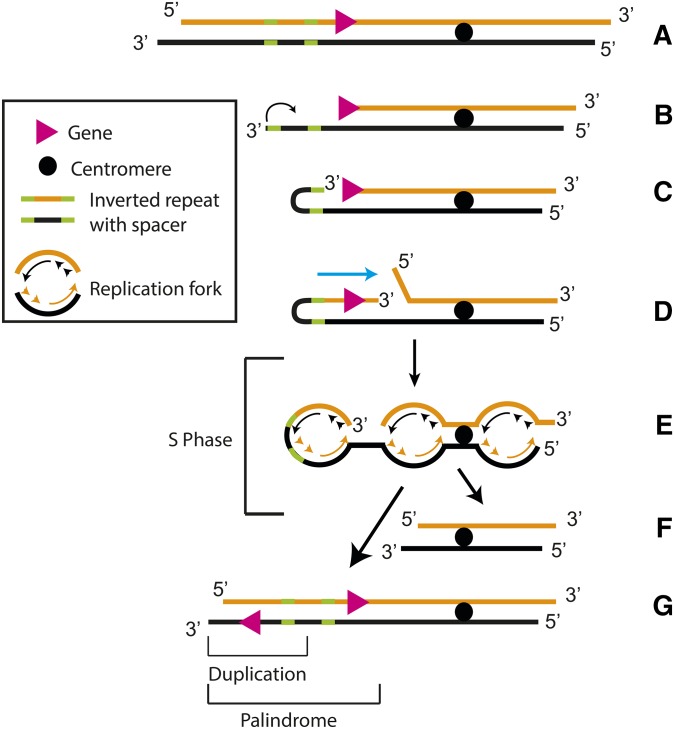
The short IR-induced DNA synthesis model of palindrome formation. (A) Model of a chromosome: one DNA strand is black and the complementary strand is orange. (B) Telomere erosion brings an IR near the end. (C) The IR folds back and undergoes base pairing, generating a short hairpin-like structure. (D) DNA synthesis (SIRIS) initiates at the IR, the 3′ end being extended to form a longer hairpin, which remains unsealed. The cyan arrow indicates the direction of SIRIS. (E) Cells enter S phase. (F) At the end of the S phase, the chromosome has been converted into two chromatids: a longer one with a palindrome at the former hairpin end and a shorter one lacking the genes present in the palindrome.

To explain the palindrome formation triggered by telomere losses, we proposed the SIRIS model, in which DNA synthesis plays the major role (Figure 6). This model improves on previously proposed PAL mechanisms (Maringele and Lydall 2004b). SIRIS initiates at DSB-like chromosome ends, unprotected by telomeres (Figure 6A). If cells continue to divide, the unprotected chromosome ends undergo progressive DNA losses, due to the end-replication problem and nuclease activities. Occasionally, degradation brings short-inverted DNA sequences [inverted repeats (IRs)] near the end of the chromosome. IRs are important for palindrome formation following telomere losses, since they are found between the palindrome arms of PAL strains (Maringele and Lydall 2004b). When the IR becomes single stranded (Figure 6B), due to the end replication problem and nuclease activity, it loops back and undergoes base pairing, thus generating a short hairpin-like structure (Figure 6C). While many short hairpins may be ignored or degraded, some may recruit DNA polymerases. If recruited, polymerases initiate DNA synthesis, *e.g.*, SIRIS at the 3′ OH end of the short hairpin, using one DNA strand (depicted in black) as a template (Figure 6D). Thus, the short hairpin is converted into a longer hairpin. The hairpin is open (or “broken”), because the newly synthesized DNA strand does not ligate with the “old” complementary strand (both depicted in orange in Figure 6D). The hairpin structure lasts until the next S phase (Figure 6E), when DNA replication converts the hairpin into a palindrome (Figure 6G). The old complementary strand is converted into a short chromatid lacking the genes involved in the palindrome (Figure 6F),

Palindromes have different sizes, depending upon how far the IR-induced DNA synthesis proceeded before cells entered the S phase. Some of the palindromes detected by CGH are surprisingly long (500 kb of duplicated DNA) and could even incorporate centromeres (Figure S1), suggesting that polymerases involved in SIRIS are very processive and most likely associated with factors unwinding and/or resecting DNA. However, PAL strains lacking the Dpb3 and Dpb4 subunits of polymerase ε were also able to proliferate long term, suggesting that these subunits are not essential for the polymerase activities leading to palindrome formation (Figure 5B). It was previously shown that Dpb3 and Dpb4 inhibit escape from senescence, acting complementary to Exo1 (Deshpande *et al.* 2011). This inhibitory effect most likely reflects their role in the “vicious circle” of replicative senescence, rather than a role in inhibiting palindrome formation. According the vicious circle model, DNA synthesis and DNA resection provide alternating substrates for different checkpoint sensors, thus preventing sensor adaptation and facilitating senescence maintenance (Deshpande *et al.* 2011).

Interestingly, we found that Rev3, which is the catalytic subunit of polymerase ζ, was essential for cells lacking telomeres to escape from senescence (Figure 5A). Its subunit Rev7 appeared to be important, but not essential, consistent with the fact that Rev7 is not essential for the activity of Pol ζ (Nelson *et al.* 1996). However Pol32, subunit of both polymerase ζ and Δ (Makarova and Burgers 2015), was not required. The smaller effect of the *pol32*∆ mutation compared to mutations in Dpb3 or Dpb4 may suggest that Pol32 has ambivalent functions during senescence. For example, by associating with polymerase ζ, Pol32 may facilitate escape from senescence (similarly to Rev7), whereas by associating with polymerase Δ, it may inhibit escape (similarly to Dpb3 and Dpb4). If both polymerases were active, the overall effect of Pol32 would be small, consistent with our observation.

There are several hypotheses explaining the essential role of polymerase ζ in PALs: (1) Pol ζ generates mutations essential for cells to escape from senescence and (2) Pol ζ is essential for SIRIS and palindrome formation. We cannot differentiate between these hypotheses at this time. Previous data showing an increase in polymerase ζ activity during senescence (generating base substitutions and frameshift mutations) would be consistent with the first hypothesis (Meyer and Bailis 2007). Further experiments are needed to understand the role of DNA polymerases during replicative senescence and palindrome formation.
